# Supplementary material for: Are carbon emissions trading and green financial instruments synergistic? -Comprehensive quantitative research based on content analysis
Source: PLoS One. 2024 Mar 7;19(3):e0298601. doi: 10.1371/journal.pone.0298601 (PMC10919601; doi:10.1371/journal.pone.0298601)
Supplement: S1 Data — (ZIP) [file pone.0298601.s001.zip › Data/地级市Z_F工作报告.zip!/╔╜╢1⁄2/╝├─╧╩╨/└┤╫╘╣1⁄2╓┌║┼í╛┬φ┐╦╩2╛▌═°í┐_2017.docx]

各位代表：

　　现在，我代表市人民政府向大会报告工作，请予审议，并请各位政协委员提出意见。

　　一、过去五年及2016年工作回顾

　　过去的五年，是济南发展历程中不平凡的五年，也是经济社会发展取得新成就的五年。五年来，特别是党的十八大以来，在以习近平同志为核心的党中央坚强领导下，全市上下全面贯彻落实党中央国务院、省委省政府和市委各项决策部署，坚持稳中求进工作总基调，主动适应经济发展新常态，自觉践行新发展理念，务实求进、迎难而上，圆满完成了十五届人大一次会议确定的目标任务。

　　五年来，综合实力大幅增强，质量效益不断提升。2016年全市生产总值达到6536.1亿元，年均增长8.8%。一般公共预算收入641.2亿元，年均增长14.6%。社会消费品零售总额达到3764.8亿元，年均增长12.2%。固定资产投资完成3974.3亿元，年均增长15.5%。与2011年相比，税收占一般公共预算收入比重达到77.7%，提高4个百分点；服务业增加值比重达到58.9%，提高5.8个百分点；高新技术产业产值比重达到43.7%，提高5.1个百分点。

　　五年来，改革开放不断深化，发展活力持续增强。以简政放权为突破口，完成新一轮政府机构改革，行政审批、商事制度、国企国资、城乡统筹等多项改革深入推进，全面实施了责任清单、权力清单，制约和阻碍发展的藩篱进一步突破，五年累计新增市场主体43.2万户。深入实施对外开放战略，积极融入“一带一路”建设，全面开展构建开放型经济新体制综合试点试验工作，累计实现进出口490亿美元、实际到账外资72.7亿美元，外贸进出口保持平稳增长。

　　五年来，城乡面貌明显改观，功能品质不断提升。新型城镇化加速推进，建成区面积由363平方公里拓展到403平方公里，户籍人口城镇化率达到63%。基础设施日趋完善，“高快一体”路网基本形成，城乡水、电、气、热、宽带等基础设施得到改善，济南地铁时代正式开启。宜居品质不断提高，荣获国家卫生城市、国家森林城市称号，建成全国首个水生态文明城市。天下第一泉景区荣获5A级景区，泉城特色更加彰显。美丽乡村标准化建设覆盖率达到42%，农村人居环境不断提升。

　　五年来，民生保障日益完善，社会事业快速发展。城乡居民人均可支配收入分别达到43052元和15346元，年均增长9.4%和10.6%。积极拓宽就业渠道，新增就业99.2万人。教育均衡发展水平不断提高，医疗服务能力持续增强，基本实现人人享有社会保险。累计开工建设保障性安居工程15.7万套，一大批住房困难家庭告别棚户区。大力实施脱贫攻坚，累计24.5万人实现了脱贫。法治济南、平安济南建设深入推进，营造了和谐稳定发展的良好局面。

　　各位代表！

　　2016年是本届政府的收官之年，是我市“打造四个中心,建设现代泉城”全面破题起势之年。全市上下在市委坚强领导下，全力推进“433”工作体系，攻坚克难，真抓实干，“四个中心”建设实现了“一年有势头”的阶段性目标。

　　（一）着力推进创新转型，促进经济提速增效。实施供给侧结构性改革、中国制造2025济南行动计划等政策措施，经济运行稳中有进、进中向好。全市生产总值增长7.8%，高于全省0.2个百分点；一般公共预算收入增长9.9%，高于全省1.4个百分点；固定资产投资增长13.7%，高于全省3.2个百分点；社会消费品零售总额增长10.4%，与全省持平；进出口增长13%，高于全省9.5个百分点；规模以上工业增加值增长7.3%，高于全省0.5个百分点；主要经济指标全部达到或超过全省平均水平。

　　强化创新驱动发展，加快推进“四个中心”建设。金融支撑作用明显增强。中央商务区建设全面启动，中国银行山东省分行等44家金融机构落户济南。新增主板、新三板上市挂牌企业59家，金融业增加值、税收占全市比重分别达到11%和13.6%。物流业增势强劲。社会物流总额达到2.13万亿元，增长12%。传化公路港等一批项目加速推进，济新欧专列开通工作启动，我市被评为2016年度中国物流业十大最具创新力城市。新经济发展迅速。电子商务交易额增长46%，生物医药、高端装备等新兴产业均增长15%以上。软件和信息服务业产值达到2351.6亿元，规模居全省首位。创新创业步伐加快。山东工研院科研孵化基地首期建成启用，浪潮思科网络科技有限公司正式运营。新增众创空间、科技孵化器40家，新增高新技术企业268家，入选山东半岛国家自主创新示范区、国家促进科技和金融结合试点城市、国家小微企业创业创新示范基地城市。着力扩大有效投资。卓有成效开展“投资促进年”和重点项目建设督查评议活动，签约落地万达文旅城等一批大项目，引进市外投资1176.6亿元，增长23.9%；实际到账外资112.3亿元，增长14.7%；新增省外签约项目合同投资额1994亿元，位列全省第一。

　　(二)着力强化载体功能，加快城乡统筹发展。城市发展框架进一步拉开。城市总体规划获国务院批复，章丘实现撤市设区，科学确定了多中心、网络型城市空间格局。基础设施建设全面提速。轨道交通3条R线全部开工，济青高铁、二环快速路等重要交通设施建设有序推进，新增普通公交、社区公交、通勤快巴等线路17条。完成水、气、热等管网建设改造380公里，新增集中供热面积800万平方米。农业农村工作不断加强。粮食总产达到51.45亿斤，实现“十四连丰”。美丽乡村建设加快推进，建成省、市级示范村117个。高标准推进农村“七改”工程，改造农村公路370公里，完成农厕改造14.9万户，基本实现城乡环卫一体化。县域经济有了新发展。一大批引领型项目在县域开工建设，园区经济、特色产业、小城镇建设成为县域发展的重要支撑，章丘跻身全国综合实力百强县29位。

　　（三）着力改善环境质量，提高生态建设水平。坚持标本兼治，推进治霾、治水、治山、增绿工作。治霾攻坚力度加大。东部老工业区搬迁改造、关停腾退工业企业40家，提前完成钢铁、煤炭产能化解任务。集中开展渣土运输、工地扬尘治理，淘汰改造建成区35蒸吨及以下燃煤锅炉，27万余户居民实现清洁能源和清洁燃煤替代。开展“啄木鸟在行动”，加大对大气污染违法违规行为的曝光力度。全年环境空气良好以上天数增加29天，从去年11月开始连续5个月退出全国74个重点城市空气质量排名后10位。治水力度加大。五库连通工程竣工，完成龙脊河等7处河道景观提升，13处黑臭水体整治完工，城市污水集中处理率达到96%以上。划定保泉“四线”，泉水博物馆建成使用，大明湖景区全部免费开放。造绿增绿力度加大。全年造林5.2万亩，建设绿地260公顷，绿化裸露土地476万平方米。26处山体公园建成开放，泉城公园改造提升项目荣获中国人居环境范例奖。环境综合整治力度加大。实施渣土运输、烧烤经营、户外广告、城区管线、垃圾收运、施工噪音集中整治，启动铁路沿线综合治理，一大批群众反映突出的城市顽疾得以解决。

　　（四）着力破解发展难题，不断扩大改革开放。深化放管服改革。削减市级行政权力81项，完成简化优化公共服务流程试点，“五证合一”、事中事后监管等改革顺利推进，3258项市级行政权力下放高新区。深化投融资体制改革。创新财政资金使用方式，组建财金控股集团、产业发展投资集团，设立各类产业投资基金30支。深化国企国资改革。44户困难企业帮扶解困基本完成，清理僵尸企业29户。深化农业农村改革。农村土地承包经营权确权登记颁证工作全面完成，农村产权交易、信息、担保三大平台基本建成，小麦、玉米、棉花政策性农业保险实现全覆盖。开放发展迈向更高水平。支持和服务企业走出去，在全国率先构建4个海外企业联盟，德国斯图加特中德中小企业协同创新中心、美国硅谷浪潮海外孵化器正式启用，对外承包工程合同额、营业额分别完成54亿和35亿美元，居全省第一和第二位。

　　（五）着力做好民生实事，持续增进民生福祉。民生投入继续增加，占预算支出的比重提高到75.2%。着力解决群众关注的难点问题，全面完成15件民生实事。坚持就业为民生之本，全国巾帼家政服务职业大赛电视总决赛在济南举行，新材料产业园获批国家级海峡两岸青年创业基地，全年新增城镇就业18.9万人，农业富余劳动力转移就业5.1万人。社会保障更加完善，全面启动全民参保登记计划，提高了职工和居民七大类社会保险待遇标准，建立了职工长期医疗护理保险制度。棚改旧改步伐加快，54个省级棚改项目4.9万套安置房全面开工。多措并举开展精准扶贫，6.4万农村贫困人口实现脱贫。努力办人民满意教育，开工新建改扩建中小学校90所、竣工55所，有效解决了大班额问题。开展文化下乡活动，全市90%的村建成农村文化大院。加快养老服务业发展，新建标准化社区日间照料中心65处、农村幸福院117处。加强平安济南建设。深入开展“法治六进”活动，市民法治意识普遍增强。强化双拥共建，实现全国双拥模范城“八连冠”。国家食品安全城市创建通过省级检查验收，食药监管、安全生产形势稳定向好。健康城市创建正式启动，群众体育蓬勃发展。持续提升为民服务平台，12345热线服务规范正式成为国家标准。人防、史志、气象、地震、慈善、红十字、民族宗教以及援藏援疆等工作都取得新的进步。

　　去年以来，政府系统坚持不懈加强自身建设，按照市委统一部署，深入开展“两学一做”学习教育，着力反对“自以为是”，围绕“快干实干”，坚持科学决策，强化责任担当，加强作风建设，狠抓督查落实，有力推动了各项工作。自觉接受人大法律监督和工作监督，严格执行人大及其常委会决定决议，主动接受政协民主监督和社会监督、舆论监督，全年办理人大代表建议271件、政协提案665件。严格落实党风廉政建设责任制，持续强化行政监察和审计监督，查处违反中央八项规定精神等问题126起，维护了政府良好形象。

　　各位代表!

　　通过全市广大干部群众共同努力，当前，我市已经形成了百舸争流、竞相发展的生动局面，大家心往一处想、劲往一处使，凝心聚力谋发展，铆足干劲抓落实，展示出蓄势待发、生机勃勃的发展前景。这是我市推进省会现代化建设步伐、率先高水平全面建成小康社会最大的优势、最强的底气。济南发展的实践充分证明，要在新一轮发展中实现弯道超车、变道换向，必须顺应泉城人民建设美好家园的新期待，坚持“打造四个中心,建设现代泉城”奋斗目标，一张蓝图绘到底，扭住目标持续用力；必须解放思想，树立敢为人先的胆识，不断自我加压，争创一流，从更高维度更大格局谋划推动济南发展，努力在保持经济中高速发展上挑大梁、在推动新旧动能转换上勇攻坚、在解决重大民生问题上做表率，力争有更多工作实现赶超，走在全省乃至全国前列；必须强化责任担当，大干、快干、苦干、实干，勇于在改革发展各项工作中敢啃硬骨头，寻找新出路，干出新天地。“艰难困苦，玉汝于成”。只要我们发扬“钉钉子”精神，汇聚众智、勇于开拓，撸起袖子加油干，“打造四个中心,建设现代泉城”奋斗目标就一定能实现。

　　各位代表!

　　济南改革发展取得的成绩来之不易。这是全市上下全面贯彻落实党中央国务院和省委省政府各项决策部署的结果，是市委统揽全局、科学决策和市人大、市政协监督支持的结果,是全市人民同心同德、艰苦奋斗的结果。在此，我代表市人民政府，向全市广大干部群众、各界人士、驻济部队、武警官兵和中央、省驻济单位，向港澳台同胞、海外侨胞和国际友人，表示衷心的感谢!

　　我们也清醒地认识到，济南正处在经济社会深刻转型、创新驱动发展的攻坚阶段，实体经济发展动力不足，新产业支撑力不强，产业结构还不够优；重点领域改革还不深入，创新创业的体制机制、政策环境仍不完善；城乡区域发展还不均衡，县域经济实力不强，空间布局不合理，城市功能仍需进一步提升；雾霾、交通拥堵、城乡脏乱差问题还比较严重，民生保障、社会治理、安全生产等方面仍存在不少短板。这些问题，我们要高度重视，下决心予以解决。

　　二、今后五年及2017年目标任务

　　刚刚闭幕的中共济南市第十一次党代会，全面谋划了我市未来五年全面小康美好蓝图。把党代会确立的目标任务落到实处是政府的职责所在，让人民生活更加美好是政府的使命担当。今后五年工作的总体要求是：高举中国特色社会主义伟大旗帜，以马克思列宁主义、毛泽东思想、邓小平理论、“三个代表”重要思想、科学发展观为指导，全面贯彻党的十八大、十八届三中、四中、五中、六中全会和十九大精神，深入贯彻习近平总书记系列重要讲话精神和治国理政新理念新思想新战略，统筹推进“五位一体”总体布局，协调推进“四个全面”战略布局，努力践行创新、协调、绿色、开放、共享的发展理念，发挥省会优势，担当省会使命，率先高水平全面建成小康社会，基本确立全国重要的区域性经济中心、金融中心、物流中心、科技创新中心地位，基本建成与山东经济文化强省相适应的现代泉城。

　　——经济更加繁荣。全面完成“十三五”规划目标任务，经济增速高于全省平均水平，主要经济指标在副省级城市位次前移。经济持续健康发展，产业迈向中高端，质量效益显著提升，发展活力竞相迸发，开放型经济新体制基本形成，金融配置功能、物流枢纽作用、创新转化能力大幅增强，成为引领区域发展的核心增长极。推进省会城市群经济圈建设发展，加快提升首位度和辐射带动力。切实把创新作为引领发展的第一动力，深入实施创新驱动战略，加快新旧动能转换，努力建成创新型城市。

　　——城乡更加协调。全面展开城市发展框架,构筑“一主一副、五个次中心、十二个地区中心、两个卫星城”组团发展格局。推进济南新区建设，打破东西带状格局，解济南发展之困，让泉城拥抱黄河，实现两岸互动、北跨携河发展,推进济南发展由“大明湖时代”走向“黄河时代”。城市功能品质显著提升，现代化国际化水平大幅提高。新型城镇化加快推进，户籍人口城镇化率提高到68%，县域经济实现跨越提升，城乡发展更趋均衡。

　　——生态更加优美。实现生产空间集约高效、生活空间宜居适度、生态空间山清水秀，打造生产美、生活美、生态美的“品质泉城”。环境质量持续改善，主体功能区布局和生态安全屏障加快形成，山水泉城特色进一步彰显。大力建设美丽宜居乡村，农村面貌显著改观。扎实创建国家生态城市和生态园林城市，努力建设天蓝、地绿、水清、泉涌的绿色泉城和生态家园。

　　——生活更加幸福。让人民群众有更满意的收入、更好的教育、更可靠的社会保障、更高水平的医疗卫生服务、更舒适的居住条件，城镇社区形成商业、医疗、文化、健身、养老等15分钟生活服务圈，新型农村社区配套更加完善，城乡居民逐步享受到均等化基本公共服务，享受到丰富多彩的精神文化生活。

　——社会更加进步。社会事业协调发展，市民文明道德建设得到加强，人民素质不断提高，文化软实力继续增强，政务服务高效清廉，法治环境更加完善，社会治理现代化水平明显提升，成功创建全国文明城市，现代泉城日益焕发自信与风采，公平正义得到保障，社会既保持和谐稳定又充满旺盛活力。

　　面对未来五年美好蓝图，我们信心满怀、枕戈待旦。只要我们紧紧围绕市第十一次党代会确定的目标任务，凝心聚力，砥砺奋进，就一定能铸造济南发展的新辉煌，人民生活一定会更加幸福美好！

　　各位代表！

　　2017年是供给侧结构性改革的深化之年，也是“打造四个中心、建设现代泉城”深入推进之年。今年经济社会发展的主要预期目标是：生产总值增长8%左右，一般公共预算收入增长9%左右，固定资产投资增长12.5%左右，社会消费品零售总额增长10.5%左右，实际到账外资增长6%左右，进出口总额增长6%左右，城乡居民人均可支配收入分别增长8%左右和8.5%左右，居民消费价格涨幅3%左右，城镇登记失业率控制在4%以内，节能减排完成省定指标。

　　为实现上述目标，我们要重点抓好以下工作：

　　（一）毫不动摇推进“四个中心”建设。深入实施三年行动纲要，全力提速增效，严格考核奖惩，确保实现“两年有看头”的工作目标。

　　加快区域性经济中心建设。全面落实稳增长政策，着力做大经济总量。强化实体经济。积极帮助企业解决生产经营难题，培植壮大骨干企业，启动主导产业规上企业新一轮技术改造，实施小微企业产业结构和治理结构融合升级“双升”战略。强化有效投资。围绕推进基础设施建设、产业更新和消费升级，大力撬动社会投资，切实发挥好重点项目带动作用。强化消费支撑。加快发展教育、文化、康养、家政、旅游、休闲等服务消费，促进本地产品提档升级。围绕留住本地消费、扩大外来消费，加快推进百联奥特莱斯、宜家等一批高端服务业项目。强化品牌建设。深入实施质量、标准、品牌战略，弘扬工匠精神，积极培育名企、名家、名品，推进国际商标注册工作，让更多济南品牌驰誉海内外。

　　加快区域性金融中心建设。聚焦重点片区。举全市之力建设中央商务区，加速绿地、平安、华润等标志性项目建设，积极创建国家级产业金融创新发展试验区。聚焦重点机构。着力引进中德住房储蓄银行、工银安盛资产管理公司等金融机构，推动齐鲁银行、济南农商银行、德华安顾等本地法人机构增强资本实力，吸引周边大企业财务公司落户我市，打造总部经济集聚区。聚焦产融结合。加强银企对接，扩大直接融资，新增主板、新三板上市挂牌企业40家、区域股权交易市场挂牌企业20家。聚焦环境营造。创新金融模式，排查防控金融风险，着力构筑一流的金融生态环境。

　　加快区域性物流中心建设。强化交通体系支撑。尽快开通济新欧货运班列，加快济南机场北指廊、绕城高速大东环等项目建设，依托京沪高铁、济青高铁、济郑高铁构建高铁网主框架，抓好济南都市圈城际铁路建设，打造“米”字型高铁网，推动小清河复航，构筑水陆空交通支撑体系。招大引强。抓好京东物流等30个重点项目建设，紧盯世界100强和国内50强物流企业，吸引更多知名企业在我市投资发展。优化布局。推进桑梓店等8个重点物流园区建设，为物流项目招商和市区专业市场外迁预留空间。壮大规模。组建物流产业投资基金，鼓励企业兼并重组，新增规模以上物流企业40家、营业收入过10亿元企业5家以上、过百亿元企业5家以上。

　　加快区域性科创中心建设。打造创新平台。抓住山东半岛自主创新示范区建设契机，加快高新区“一区两城两谷”建设，推进国家重大新药创制平台、量子通讯研究院等平台建设，支持浪潮与思科深度合作，争创网络技术国家实验室，新增省级以上企业研发机构30家。强化校企、校地合作。及时解决驻济高校、科研院所发展中遇到的难题，完成山东大学主校区规划调整、征地拆迁等工作，进一步完善长清大学城配套服务。促进产学研深度融合。搭建泉城科创交易大平台，加强知识产权保护运用，争创国家科技成果转移转化示范区。创新科技金融服务体系，推广应用科技创新券。大力实施“泉城双创”人才计划。深化人才管理体制改革，优化人才服务保障机制，完善人才入境、落户、安居等政策。完善科技奖励制度，鼓励自主创新，加快集聚高层次创新创业人才和领军团队，鼓励科研人员携成果创业、带技术加入企业创新发展，让科研人员创新价值合理合法得到回报，让更多千里马竞相奔腾，让更多天下英才创业泉城、圆梦济南。

　　(二)毫不动摇推进五项重点工作。五项重点工作既是稳增长的关键，又事关城市环境形象提升，必须聚力攻坚，加快推进。

　　强力推进项目建设。抓好总投资8200亿元240个市级重点项目建设，年计划投资2100亿元。坚持大抓项目、抓大项目，强化全程服务，加快推进西部会展中心等一批在建项目投产运营；集中优势资源，加快推进华谊兄弟电影城等一批签约项目开工建设；加强策划规划，加快推进绿地启迪科技城等一批重大项目签约落地。优化再造绿色通道，全面推行容缺办理、并联审批，对市级重点项目实施全过程无偿代办。加快济南国际医学科学中心建设，高水平完成占地35平方公里的片区规划，加快村庄整合，推动山东第一医科大学主校区、肿瘤专科中心等一批项目开工建设，积极申报国家医疗健康产业综合试验区,努力把济南打造成国际性的医学高地，为全民健康提供优质的医疗服务资源。

　　强力推进招商引资。开展“投资提升年”活动，努力引进一批“高大新特优”项目，确保引进市外投资增长15%以上。在产业链招商上求突破。按照“抓龙头、铸链条、建集群”的思路，成立十大产业招商组，发挥重汽、浪潮、圣泉、九阳、韩都衣舍等骨干企业作用，围绕提高本地配套率、延伸产业链招商引资，壮大优势主导产业。在发挥省会优势招商上求突破。充分挖掘省会行政、科教、人才等资源优势，争取更多项目、资金和政策支持。发挥“四个中心”建设扶持政策引导作用，瞄准世界500强、国内500强，集聚更多优质产业项目。在重大活动招商上求突破。充分利用新加坡—山东经贸理事会、香港—山东周等主题活动，策划组织专业招商推介，开展精准招商。在形成招商合力上求突破。坚持引资与引智并重，拓展社会化招商、以商招商新渠道，建设专业招商队伍，强化考核奖惩，促进招商引资取得新成效。不断加强营商环境建设，努力打造审批最少、服务最优、效率最高的投资环境，切实提高区域发展竞争力。

　　强力推进棚改旧改。为让更多市民尽快改善居住环境，今年要集中攻坚重点片区、危旧厂区、城区孤岛等范围内棚改旧改存量，新开工华山北、新东站片区等53个棚户区改造项目，确保完成6.49万套棚改安置房开工任务。优化协调推进机制，强化棚改旧改全市统筹，完善棚改旧改、房屋征收政策和管理实施体制，建立房屋征收信息平台，切实维护群众合法权益。加快老旧住宅小区整治提升，完善治安防控、环卫消防、水电气暖等硬件设施，启动既有住宅增设电梯工作，扩大物业管理覆盖面，完成278个老旧小区整治改造任务，努力让市民在“干干净净、整整齐齐”的环境中居住生活。

　　强力推进拆违拆临、建绿透绿。今年以来，按照“先政府后个人、先党员干部后群众”的要求，集中开展了拆违拆临、建绿透绿专项工作，根除了一批积存多年的违法违规问题，切实维护了公平正义，受到广大市民力挺点赞。下一步要继续强力攻坚，依法依规，8月底前基本完成确定的违法建筑拆除工作。坚持还绿于民、还空间于民，将腾出的土地主要用于绿地建设，做到即拆、即清、即绿，年内新增绿地220万平方米、绿化覆盖率达到40%以上，让市民共享绿色福祉。

　　强力推进全国文明城市创建。按照“为民、靠民、不扰民”的理念，突出重点区域，改善城市面貌，打造“平、亮、绿、美、净、齐”的城市环境，努力实现“城市让生活更美好”。全面提升城市管理水平，着力推进城市管理网格化、精细化、智慧化、法治化，为人民群众提供精细的城市管理和良好的公共服务。开展城市管理整治行动，加强对城区杆线、户外广告、垃圾死角、违规经营、乱停乱放、乱贴乱画的治理，每一季度通过第三方评估一次，切实奖优罚劣，下大气力解决脏乱差问题，彻底消除杆线“蜘蛛网”等视觉污染。深入推动313公里铁路沿线环境整治，打造大纬二路等6条示范样板街。深化窗口文明创建，深入实施“2017年创城百项主题活动”，动员社会各界以主人翁姿态积极投身创建工作，形成人人参与的文明城市创建新局面。

　　（三）毫不动摇打好三大攻坚战。把治霾、治堵、脱贫作为当前最大的民生，坚持不懈，持续用力，力争尽快抓出成效。

　　坚决打好蓝天保卫战。继续实施大气污染治理十大措施，强化精准治霾，推进扬尘、燃煤、工业、机动车尾气、油烟和烧烤污染治理。严格煤炭总量控制，淘汰市域范围内35蒸吨以下燃煤锅炉，35蒸吨以上燃煤锅炉全部实现超低排放，实施散煤替代工程，加快推进“外热入济”。加大建筑工地扬尘治理力度，关闭城乡规划区内全部露天开采矿山。强化工业污染源治理，按时完成济钢等东部老工业区16家企业停产搬迁。大力推广新能源汽车，新建住宅配建的车位100%建设充电设施或预留充电设施建设安装条件。推进区域大气污染联防联控，实施环境网格化监管，严格路长责任制，确保各项减排限排措施落实到位，努力让泉城的蓝天一天天多起来。

　　持续推动治堵攻坚。深入实施交通治堵十大措施。推进城市综合交通体系规划建设，实施二环线匝道周边道路畅通综合治理，打通刘长山路西段等27条丁字路、瓶颈路。落实公交优先战略，优化公交网络、加密线路班次，推进龙洞等公交场站枢纽建设，继续开辟高峰线路、通勤快线、社区公交、区间公交，加快现代无轨电车网建设。深化交通管理综合整治，加快智慧交通建设，优化改造拥堵路段，集中整治交通违法，畅通道路微循环，科学配置施工区域道路资源，出台停车场建设扶持政策，规划建设慢行系统，鼓励市民绿色出行，让出行更加便捷顺畅。

　　基本完成脱贫任务。要以“绣花”的功夫做好精准扶贫，整合力量、加大投入，年内完成剩余1.56万贫困人口脱贫任务。强化产业扶贫，实施711个产业扶贫项目，鼓励扶贫工作重点村大力发展特色种养加、乡村旅游、农村电商，增强“造血”功能。积极推广金融扶贫，加快推动政府贴息、担保、风险补偿等配套政策落地实施。全面做好保障兜底脱贫，重点推进医疗精准扶贫。扎实做好脱贫巩固，对已脱贫人口保持政策力度不减。定期开展集中督查和重点专项检查，确保精准扶贫政策措施落实到位，防止假脱贫、“被脱贫”、数字脱贫，稳定实现农村贫困人口不愁吃、不愁穿，义务教育、基本医疗和住房安全有保障。扎实做好与湘西州、临沂市结对扶贫协作工作，确保完成年度帮扶任务。

　　（四）毫不动摇推进产业转型发展。坚持高端高质高新方向，实施先进制造业和现代服务业双轮驱动，打造经济增长新动力。

　　加快壮大省会产业集群。全面推动传统产业改造提升，在此基础上，集聚优势资源，实施聚焦突破，突出发展大数据与新一代信息技术、智能制造与高端装备、量子科技、生物医药、先进材料、产业金融、现代物流、医疗康养、文化旅游、科技服务十大产业，加快规模扩张和提质增效，构建上中下游密切衔接、配套完善的现代产业集群。到2020年，十大产业全部达到千亿级产业规模，智能制造与高端装备产业达到两千亿级，大数据与新一代信息技术产业达到五千亿级；主营业务收入过千亿元企业达到2家，过百亿元企业达到20家以上，“专精特新”中小企业达到300家以上。我们要通过努力，使省会的产业层次更高、综合效益更好、核心竞争力更强。

　　加快培育新兴产业。加快大数据产业发展。制定实施《数创公社发展规划》，打造济南数据科学中心，争创国家级大数据综合试验示范区和国家新型智慧城市试点。发展量子科技。筹建量子通讯科技园，打造量子通讯创新服务平台，建成全国首个应用型量子通讯专网。发展智造经济。实施《济南市智能制造产业五年发展规划》，加快高端数控机床、机器人、智能制造专用装备、智能仪器仪表及控制系统等产业成长，力争5年培育50家智能装备重点骨干企业。发展地铁经济。做好基础建设、装备制造、电器设备、公共运营全产业链谋划，设立产业发展基金，筹建轨道交通装备制造集团，打造轨道交通装备产业园。加快技改提升，实施60个亿元以上技改配套项目，抓好齐鲁制药、力诺等一批企业数字化车间建设，推动重汽小镇、阿胶小镇建设，加快提升装配式建筑、电力设备等产业系统集成能力，着力提高产业附加值。大力推动民参军、军转民和军民资源共享，重点发展特种汽车、特种计算机、航空电子、新材料等产业，争创国家级军民融合产业发展基地。

　　加快发展现代服务业。推进生产性服务业高端化、生活性服务业品质化，现代服务业比重提高到55%。以万达文旅城、济南国际医学科学中心、蓝海领航、市中工业设计产业区等项目为重点，打造一批服务业集聚区，大力发展以工业设计、检验检测、科技服务为代表的生产性服务业，以休闲旅游、文化体育、医疗健康为代表的生活性服务业，以总部经济、现代金融、会展服务为代表的高端服务业。加速推进楼宇经济做大规模、做优产业、做强特色，继续打造一批税收亿元楼。加强旅游基础设施建设，整合提升旅游资源，启动创建国家全域旅游示范市。加快发展分享经济，推进实体零售创新转型。健全商贸流通体系，抓好历下区国家服务业综合改革试点工作，建设经纬巷等一批特色街区，推动城区专业批发市场有序外迁、整合提升。

　　加快发展现代农业。深入推进农业供给侧结构性改革，加快培育农业发展新动能。优化产业结构。大力发展精致农业，推进10个万亩粮食绿色增产示范片建设，发展特色经济林、种苗花卉、中药材、食用菌、茶叶等特色高效产业。推动产业融合发展。以建设现代农业综合体为抓手，加快互联网+农业“十大平台”建设，推动“新六产”发展，开展农产品加工业示范县、示范园区、示范企业创建工程。支持农信社、粮食、供销、邮政等系统发挥优势，建立农业社会化综合服务平台。提高农产品质量。积极实施“菜篮子工程”，着力增加绿色优质农产品供给，完善从田间到餐桌全过程监管，确保农产品质量抽检合格率98%以上。激活农村要素市场。落实承包土地“三权分置”办法，建设涵盖农村全要素的产权交易市场。开发更多涉农金融产品，让农民贷款更便利更容易。通过持续推进农村改革发展，努力让农业成为有奔头的产业，让农民成为体面的职业。

　　（五）毫不动摇推进城乡统筹协调发展。抢抓国家新型城镇化综合试点机遇，统筹做好城市规划建设管理，整体提升城市承载能力和功能品质。

　　优化城市空间布局。按照组团式城市发展框架，调整完善土地利用总体规划，开展城市空间发展战略研究，启动新一轮城市总体规划修编，统筹产业、交通、基础设施和公共服务设施规划布局。加大济南新区申报工作力度，完成携河发展空间规划、黄河桥隧规划，适时启动济泺隧道、齐鲁大桥、凤凰大桥等跨河通道建设，加快崔寨片区和新材料产业园产城融合示范区建设。推动济阳撤县设区，加快章丘、长清两个卫星城建设。

　　提升泉城特色品味。建设中央泉水区。加快大明湖风景区功能提升，实施“一湖一环”景观亮化工程，打造“泉城夜宴”。加快泉水申遗，推进泉水“先观后用”，完成泉水直饮试点工程，开展名泉保护及河湖水系规划研究，打造章丘泉水生态标志区。建设一批亲水乐水休闲娱乐设施，办好第五届泉水节。充分利用地热资源，提升“中国温泉之都”品牌。传承好老城肌理和城市文脉。完成历史文化名城和芙蓉街—百花洲等3处历史文化街区保护规划，实施明府城、老商埠区保护开发市级战略，启动以“一园十二坊”为重点的老商埠区建设，进一步焕发老城人文魅力。

　　补齐基础设施建设短板。加快轨道交通3条R线建设，开工M2、M3线，做好轨道交通向周边县区延伸规划。启动北园高架西延、二环东高架北延建设，确保二环快速路、工业北路快速路建成通车，5年内建成市域快速交通路网。加快农村改路、改电、改校、改房、改水、改厕、改暖“七改”工程，确保2017年村村通动力电，改造厕所20万户，农村危房改造达到七级设防等级，城乡高速宽带有线网络和无线网络全覆盖，农村生产生活条件明显改善。

　　加快提振县域经济发展。坚持多予少取放活，加强顶层设计和全市统筹，推动重大战略、产业发展、基础设施向县域布局。加快开发区改革创新，在审批、土地、项目、人才、融资等方面放权让利，突破瓶颈，支持共建跨区域合作园区。设立县域经济发展基金，大力扶持特色主导产业，国家级园区更加重视引大做强，省级园区更加重视做特做优，培育一批有成长潜力的中小企业。加大财政转移支付，提升县域公共服务水平。加强县城和小城镇建设，抓好刁镇、马山、玫瑰等6个小城市培育和特色小镇建设。强化乡村连片治理，实现420个村达标升级，全面提升美丽乡村标准化建设水平。

　　(六)毫不动摇推进重点领域改革。坚持把改革作为跨越发展的强大动力，善始善终、善做善成，加快基础性关键性改革，进一步激发市场活力和社会创造力。

　　深入推进供给侧结构性改革。加大“三去一降一补”工作力度。有序推动化工行业转型升级、钢铁行业去产能和产能替补，妥善做好职工分流安置和企业债务处理工作，力争到2018年，炼油、水泥行业产能利用率提高到80%以上。坚持“房子是用来住的”，加强房地产调控政策落实，多措并举降低库存，促进房地产市场平稳健康发展。出台《进一步促进资本市场发展的实施意见》，加大规模企业规范化股份制改革、上市挂牌、债券融资支持力度，降低企业杠杆率。打好减税、降费等“组合拳”，使企业减负轻装上阵，形成竞争新优势。通过深入推进“三去一降一补”，着力构建与省会需求结构相匹配的供给体系。

　　深化“放管服”改革。以更有力的举措推进简政放权，再取消、下放、调整一批“含金量”高的审批事项，在工程建设领域实行多评合一、联合验收等新模式。深化商事制度改革，实行多证合一，推进注册登记便利化。完善事中事后监管，积极推进失信联合惩戒机制。推进互联网＋政务服务，加快建成互通共享的网上政府服务平台，努力让信息多跑路，让群众少跑腿。

　　深化财税体制改革。着力培植壮大财源，加大税源管控和开源增收力度。健全公共财政体系，逐步推进县域间民生政策保障标准、农业转移人口市民化待遇、机关事业单位工资性收入政策、运转经费保障标准、基本公共服务设施建设标准“五个基本统一”。合理划分财政事权和支出责任，推进跨部门资金统筹使用，完善跨年度预算滚动平衡机制，全面推进预决算信息公开。

　　深化投融资改革。发挥财政资金杠杆放大作用，壮大产业引导基金和股权投资基金。加快市场化融资步伐，拓宽公私合作领域及融资渠道，继续实施一批PPP项目。进一步放活机制，整合重组市属投融资公司，加快机关事业单位经营性资产改革，推动有条件的公司整体上市，提升服务城市建设和产业发展的能力。

　　深化国企国资改革。以推进经营性国有资产统一监管为突破口，分类分层深化国企国资改革，持续增强国有经济活力。不断完善公司治理结构，深化企业内部三项制度改革，加快解决企业办社会职能和历史遗留问题，提高精细化管理水平。

　　（七）毫不动摇保护改善生态环境。牢固树立“绿水青山就是金山银山”的理念，科学施策、标本兼治、且行且惜，让碧水蓝天永驻泉城。

　　提升泉城水环境。加大治水净水力度，加快雨污分流建设，实施四大污水处理厂连通联调，建设一批分散式中水处理站，全部消除黑臭水体。抓好工业污染源和农业面源污染防治，确保废水达标排放。全面落实河长制，统筹推进小清河等71条河道治理管护，实施卧虎山、锦绣川水库等水源地环境综合整治，杜绝污水直排和垃圾倾倒，确保水源地水质清洁。加大节水保泉力度，严格地下水管理，严厉打击乱开乱采行为。提升重点泉池景观，建设白泉湿地公园、华山湖公园、北湖公园。推进白云平原水库等一批水源工程，完成东湖水库输水工程、五库连通延伸工程，实现全市水资源“一张网”，统筹调用、丰枯调剂、互联互通。

　　提升山体生态水平。山泉河湖城相映生辉是济南的特色禀赋。做好“山”的文章，推进“青山入城”，是广大市民的殷切期盼。加快山体绿化，修建山间道路，安装休闲设施，推进智慧景区建设，年内建设山体公园8处、郊野公园4处，完成山体绿化提升7处、造林绿化5万亩，到2020年完成126座山体绿化，为市民休闲健身创造更好条件。

　　提升重点保护区生态功能。加强南部山区、西部湿地、北部黄河滩区生态保护，让良好的自然生态环抱济南。南部山区坚持共抓大保护、不搞大开发，划定保护“红线”，推进多规合一规划编制，完善生态补偿机制。全面提升仲宫、柳埠、西营三镇环卫设施，实施三川干道增绿添彩工程，启动复兴生态示范区建设，打造省城后花园。济西湿地坚持多做减法、少做加法，加快完善配套设施，实现年中开放运营。高标准做好玉符河生态规划，抓好沿岸乱搭乱建治理和绿化建设，打造优美的河系景观带。北部黄河滩区要逐步恢复原有生态环境，建设集生态保护、蓄水净水、风沙防治、旅游休憩于一体的黄河百里湿地森林公园，把黄河打造为城市的玉带，在北部形成一道天然绿色屏障。

　　(八)毫不动摇扩大对内对外开放。牢固树立开放发展的理念，努力讲好“济南故事”，推动更高水平更深层次对外开放。

　　营造开放发展新环境。全面推进构建开放型经济新体制综合试点试验工作，确保如期完成9大类125项创建任务。探索促进国际投资和贸易发展新方式，复制推广自贸区、国家服务贸易创新发展试点等政策，进一步扩大制造业和服务业对外开放。加大洲际直航力度，新增洲际航线2条，解决往来难问题。加快推进济南国际学校建设，解决外籍子女入学难问题。实施法制化营商环境等改革新举措，不断提升我市开放发展的广度和深度。

　　培育外贸竞争新优势。深度融入“一带一路”，加快中欧班列综合服务体系建设，推进通关便利化，优化进出口结构，促进对外经贸人文交流，构筑外贸经济新版图。坚持“引进来”与“走出去”相结合，推进中德双向协同创新中心和中德科技园建设，拓展济南海外企业联盟，鼓励骨干企业积极开拓海外市场，推动装备制造、科技服务、临空经济等产业开放发展。加快综合保税区建设，支持一达通等平台更好发挥作用，让更多的济南产品和中小企业走向世界。

　　拓展国际合作新平台。加快提升我市国际化水平，实施《济南城市国际化建设行动纲要》及2017-2018行动清单，以友城为平台促进城市公共外交。高质量办好首届市长国际咨询委员会发展论坛，借“外脑”引“外智”，不断提升济南开放发展水平。加快“侨梦苑”建设，打造华侨华人创新创业、集聚海外优质资源的高端基地。举办中德中小企业合作交流会、国际泉水文化景观城市联盟年会、中国（山东）国际医疗健康高峰论坛、济南产业金融峰会等活动，为互通有无、加强交流创造多维空间。

　　推动民营经济新发展。在大力推动对外合作的同时，对内要激活各类发展主体活力，特别要大力推进民营经济发展，实施民营经济提升工程，落实好促进民营经济发展各项政策措施，真正做到非禁即入，力争民间投资年内增长10%以上。加快中小企业服务体系建设，健全领导干部联系非公有制企业制度，支持民营企业建立现代企业制度。依法保护企业家财产权和创新收益，培养壮大企业家队伍，稳定民营企业家信心，满腔热情支持企业家专心创新创业，关心、尊重、爱护和服务企业发展。

　　（九）毫不动摇保障改善民生。牢固树立“以人民为中心”发展理念，努力提供更多更好的公共服务，今年要扎实做好为民16件实事，让群众不断感到发展有新变化、生活有新奔头。

　　深入实施就业优先战略。始终把就业放在民生工作的突出位置，千方百计增加就业岗位，全年城镇新增就业10万人，农村转移劳动力就业5万人。搭建创新创业服务平台，扩大创新创业政策扶持范围，探索“短平快”融资模式，推动大学生、返乡农民工、科技人员等重点群体创业。完善终身职业培训体系，促进老工业基地搬迁改造分流人员、农村富余劳动力、被征地农民和城镇困难群体就业，确保零就业家庭动态清零。促进社会保障公平可持续。深化机关事业单位养老保险制度改革，完善职工医疗保险制度和居民大病保险政策，扩大合规医疗费用范围。完善医保经办机构与医疗机构付费机制，降低参保人负担。稳步提高退休人员基本养老金和居民医疗保险财政补助标准，将居民基础养老金标准提高到每月110元。

　　提升公共服务水平。推进基础教育综合改革，启动中小学校长职级制改革，完善教师交流轮岗常态化机制。新建改扩建77所中小学校，全面解决城镇普通中小学大班额问题。支持和促进民办教育发展，努力提供多元优质的教育资源。深化公立医院综合改革，完善分级诊疗制度，推动市中医院东院区、市传染病医院迁建工程开工建设。适应实施全面两孩政策，优化计划生育管理服务。做好城乡孤困儿童保障和农村留守儿童关爱工作。加强文化遗产保护利用，实施城子崖考古遗址公园等20个项目，启动济南博物馆新馆、儒学博物馆、方志馆、济南现代影像数据中心、市县区档案馆建设。积极推进健康城市创建，广泛开展全民健身，举办龙舟赛、登山节等丰富多彩的活动，使更多泉城市民享受运动快乐、拥有健康体魄。

　　积极创新社会治理。坚持把社会治理重心落到城乡社区，完善社区便民服务功能，创建全国社区治理和养老服务创新实验区。大力提高12345热线接听率、办结率、回复率，使之真正成为党委政府与市民的连心桥。建设阳光民生救助体系，使困难群众遇急有助、遇难有帮，让社会充满关爱和温暖。狠抓重点领域整治、隐患排查治理，确保安全生产形势稳定向好。改进监管方式，强化源头治理，创建国家食品安全示范城市，让市民饮食更加健康、更加放心。深入推进平安济南建设，坚持打防管控治多措并举，确保社会安定和谐。深入落实“七五”普法规划，争创全国法治城市。加强国防动员，启动新一轮全国双拥模范城创建工作。继续做好仲裁、老龄、残疾人、应急管理、民族宗教、哲学社会科学等工作。

　　三、全面加强政府自身建设

　　打铁还须自身硬。全市改革发展的艰巨任务，对政府自身建设提出了更高要求。全市各级政府及其工作人员必须牢固树立“四个意识”，着力提高政治站位和政治觉悟，坚决维护习近平总书记的核心地位，维护党中央权威，维护党的团结和集中统一，以人民更加满意为目标，立党为公、执政为民，旗帜鲜明讲政治，脚踏实地干实事，努力建设责任、法治、高效、廉洁的人民政府，更好为泉城人民服务。

　　（一）强化宗旨意识，建设责任政府。要把群众的安危冷暖时刻放在心上，多办惠民生的实事，多解决群众和企业牵挂的问题。开展减证便民专项行动，最大程度便利群众。积极推进决策科学化民主化，重视发挥智库作用，广泛征求群众意见，把政府决策过程变为汇民意、聚众智、凝共识的过程。坚持夙夜在公，勤勉工作，以“食不甘味、寝不安席”的紧迫感，及时回应群众在环保、医疗、教育等方面的热切期盼，全力做好促发展、惠民生各项工作，切实当好人民公仆。

　　（二）严格依法行政，建设法治政府。落实法治政府建设实施纲要，提升各级政府和公务员队伍法治意识和社会治理能力。推进行政执法体制改革，推动跨部门综合执法和执法力量向基层下沉，建立部门执法责任清单。全面推行“双随机、一公开”监管工作，切实解决一些领域检查任性、执法扰民、执法不公、执法不严等问题。自觉接受人大法律监督、工作监督和政协民主监督，认真办理人大代表建议和政协委员提案，定期向人大报告工作、向政协通报情况。全面推进政务公开，主动接受社会监督和舆论监督，让权力在阳光下运行。

　　（三）狠抓工作落实，建设高效政府。广大干部要强化担当精神，闻鸡起舞，登高望远，保持奋发有为、力争上游的精气神，努力以国际视野、现代思维来谋划推动省会工作。坚持“实在实干实绩”导向，完善正向激励和容错纠错机制，为担当者担当，让实干者实惠，使雷厉风行、快干实干成为本届政府作风建设的鲜明特征。改革已经进入攻坚期和深水区，面对越来越多的复杂矛盾和敏感问题，要更加尊重市场规律，努力提升专业素养，以创新办法和开放思路啃掉“硬骨头”。强化工作督查落实，重点工作任务要铆紧各方责任，确保不折不扣落地抓实。严格公务人员绩效管理考核，严厉整肃懒政、怠政、不作为、推诿扯皮等行为，全面提高政府公信力和执行力。

　　（四）着力反腐倡廉，建设廉洁政府。压紧压实政府系统各级党组织主体责任，严肃党内政治生活，强化党内监督，落实监督执纪“四种形态”，层层传导压力，推动全面从严治党向基层延伸。加强党风廉政教育，严格落实中央八项规定精神，一以贯之纠正“四风”。坚守节用裕民的正道，压减年度一般性支出10%，把每一笔钱都花在明处、用在实处。充分发挥审计监督作用，严格监管公共资金、公共资源、国有资产的使用和效益。始终把纪律和规矩挺在前面，坚持用制度管权管事管人，坚定不移惩治腐败，自觉为营造风清气正的政治生态履职尽责、作出贡献，努力实现干部清正、政府清廉、政治清明。

　　各位代表，蓝图已绘就，奋进正当时。让我们紧密团结在以习近平同志为核心的党中央周围，在中共济南市委坚强领导下，不忘初心，砥砺前行，努力以新作为开启新征程、以新担当催生新发展，推动济南各项事业不断取得新成就，为“打造四个中心，建设现代泉城”作出新的更大贡献，以优异成绩迎接党的十九大和省第十一次党代会胜利召开！
